# Improvement of Depth Profiling into Biotissues Using Micro Electrical Impedance Spectroscopy on a Needle with Selective Passivation

**DOI:** 10.3390/s16122207

**Published:** 2016-12-21

**Authors:** Joho Yun, Hyeon Woo Kim, Jong-Hyun Lee

**Affiliations:** 1 Department of Biomedical Science and Engineering, Gwangju Institute of Science and Technology (GIST), Gwangju 500-712, Korea; joho@gist.ac.kr (J.Y.); cs8do@gist.ac.kr (H.W.K.); 2 School of Mechanical Engineering, Gwangju Institute of Science and Technology, Gwangju 500-712, Korea

**Keywords:** electrical impedance spectroscopy, hypodermic needle, interdigitated electrodes, selective passivation, depth profiling, biotissues

## Abstract

A micro electrical impedance spectroscopy (EIS)-on-a-needle for depth profiling (μEoN-DP) with a selective passivation layer (SPL) on a hypodermic needle was recently fabricated to measure the electrical impedance of biotissues along with the penetration depths. The SPL of the μEoN-DP enabled the sensing interdigitated electrodes (IDEs) to contribute predominantly to the measurement by reducing the relative influence of the connection lines on the sensor output. The discrimination capability of the μEoN-DP was verified using phosphate-buffered saline (PBS) at various concentration levels. The resistance and capacitance extracted through curve fitting were similar to those theoretically estimated based on the mixing ratio of PBS and deionized water; the maximum discrepancies were 8.02% and 1.85%, respectively. Depth profiling was conducted using four-layered porcine tissue to verify the effectiveness of the discrimination capability of the μEoN-DP. The magnitude and phase between dissimilar porcine tissues (fat and muscle) were clearly discriminated at the optimal frequency of 1 MHz. Two kinds of simulations, one with SPL and the other with complete passivation layer (CPL), were performed, and it was verified that the SPL was advantageous over CPL in the discrimination of biotissues in terms of sensor output.

## 1. Introduction

EIS (electrical impedance spectroscopy) devices have been widely employed in biological studies, such as single cell analysis [1,2,3,4,5,6,7,8,9] and virus detection [10,11,12,13,14,15]. In particular, many studies have reported significant differences in electrical impedance between dissimilar cells or tissues from various organs [16,17,18,19,20,21,22,23,24,25]. In the biotissue-related studies, although it is important to obtain the electrical characteristics of biotissues along with the depth, the design of the devices has not been conducive to detecting the completely buried endophytic tumors or to estimating the tumor depth from the organ surface. The micro EIS device should be designed to show sufficient accuracy in depth profiling and penetrate the biotissues easily for practical bio-applications such as tissue sampling (biopsy), surgery (laparoscopy and partial nephrectomy), and drug delivery. Inspection of the effective sensing area at the microscale will be advantageous in terms of obtaining an accurate location of target tissues in the body, and/or precise resection of tumors, with minimum positive surgical margin (practically less than 2 mm). The microscale of EIS also enables accurate drug delivery into narrow blood vessels or into the spinal cavity.

A hypodermic needle is widely used in hospitals for the aforementioned purposes and its shape can be regarded suitable for depth profiling of biotissues. A micro electrical impedance spectroscopy-on-a-needle (μEoN), which is a needle combined with an electrical impedance sensor on the needle tip, was introduced to locate the boundaries between dissimilar tissues [26]. However, the sensing electrodes, completely covered by the passivation layer, created unwanted signals from the connection lines that seriously influenced the sensor output. The measurement error increases as the length of the connection lines immersed in the biotissues increases with the penetration depth. As a result, a post-compensation process is necessary to exclude the measurement error from the sensor output, which hinders real-time measurement using the device. All of the electrodes, including the sensing electrodes, were uniformly and completely passivated by Parylene C (a low dielectric material) so that the electrodes and passivation layers were durable in terms of peeling failure while penetrating the biotissues. However, when the conductivity of the sample is much higher than that of Parylene C, the complete passivation layer (CPL) tends to confine electric fields within the passivation layer though sufficient electric fields need to pass through the sample. This makes the EIS less sensitive to the sample characteristics because the large impedance caused by CPL is connected in series with the sample resistance and capacitance.

The sensor output also considerably varied as the needle penetrated even in a homogeneous sample because of the undesirable electrical field induced by the connection lines immersed in the biotissues. Thus, the penetration depth of the needle should be held constant to ensure consistency in the sensor output. This can be a critical limitation to practical applicability in real trials where depth profiling of biotissues is needed. For applications, such as tissue/blood sampling, drug delivery, laparoscopic surgery, and partial nephrectomy, the influence of connection lines on the sensor output should be minimized by selective passivation so that the electrical impedance can be more accurately obtained at a target position in the biotissues. Thus, only the sensing IDEs (interdigitated electrodes) should be selectively exposed to the biotissues to improve the performance of depth profiling. As a result, the influence of the connection lines with SPL on the sensor output can be minimized because the contribution of the connection lines will relatively decrease compared to that of the connection lines with CPL.

To expose only the sensing IDEs to the sample with no peeling, a selective passivation layer (SPL) with strong adhesion between the IDEs and the insulation layer are required. Unlike previous studies, which employed Parylene C as an insulation and passivation layer, silicon dioxide (SiO_2_) was employed for insulation layer because of its strong adhesion with electrodes compared to Parylene C. Then, SU-8 photoresist was employed as the selective passivation material that can be precisely patterned with the photolithography process. SU-8 also features excellent chemical resistance, strong mechanical properties, and biocompatibility after enough hard baking [27,28].

The newly developed μEoN-DP (μEoN for depth profiling), which can measure the electrical impedance enables depth profiling of biotissues in real-time; thus, the target position can be located by analyzing the measured electrical impedance between dissimilar tissues. As a preliminary experiment, the discrimination capability of the μEoN-DP was evaluated using PBS (phosphate-buffered saline) at the various concentration levels. Then, the main experiment for depth profiling was conducted to discriminate dissimilar tissues while the μEoN-DP penetrates the four-layered (fat_1_–muscle_1_–fat_2_–muscle_2_) porcine tissues. Finally, two types of 3D COMSOL simulations (COMSOL, Inc., Burlington, MA, USA) were carried out to verify the advantage of SPL over CPL in terms of the sensor output: a) only the sensing IDEs with SPL were exposed to biotissues; and b) the whole electrodes (IDEs and connection lines) with CPL were exposed to biotissues.

## 2. Materials and Methods

### 2.1. Device Design

**Figure 1:**
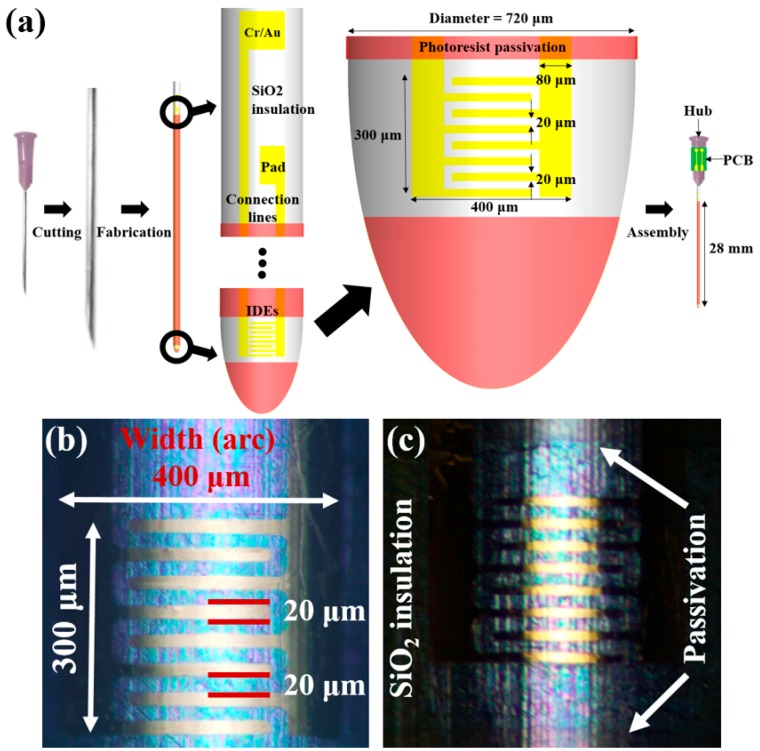
(**a**) Schematic design of the μEoN-DP (micro electrical impedance spectroscopy-on-a-needle for depth profiling); photo images of the fabricated IDEs on the curved surface of the needle; (**b**) overall length and width of the IDEs were 300 μm and 400 μm, respectively; both the width and gap of the IDEs were as small as 20 μm; and (**c**) selective passivation layer (SPL) on the needle insulated by SiO_2_.

A micro electrical impedance spectroscopy-on-a needle for depth profiling (μEoN-DP) was newly designed, as shown in Figure 1a. In the case of μEoN having a CPL on the electrodes, the connection lines caused measurement errors as the penetration depth of electrodes increased. Thus, the passivation layer of the μEoN-DP was selectively removed on the sensing area to increase the ratio of impedance measured from the connection lines to that measured from the sensing IDEs, which enables the sensing IDEs to predominantly contribute to the measurement. As a result, the electrical impedance of a sample can be measured at a specific position, minimizing the influence of immersed connection lines on sensor output.

Strong adhesions are required between the needle surface, insulation layer, electrodes, and passivation layer in order to enhance the durability of μEoN-DP. Adhesion between the needle and the insulation layer of Parylene C proved difficult to enhance, although the adhesion between the insulation layer of Parylene C and sputtered electrodes could be improved using the reported techniques [29,30,31]. The passivation layer of Parylene C cannot ensure reusability for a number of experiments because of the low mechanical properties. Hence, silicon dioxide (SiO_2_) and SU-8 photoresist were selected through a durability test as the materials of the insulation layer and SPL, respectively.

The length of the hypodermic needle used in this study was 28 mm, which is longer than that used in previous studies (20 mm), to measure the electrical impedance at positions deeper within the biotissues. The length of the IDEs was designed to be as small as 300 μm, considering the dimensions required for clinical applications, such as small tissue biopsy, drug delivery through blood vessel, or spinal cavity, and partial nephrectomy with less positive surgical margin.

### 2.2. Device Fabrication

The fine electrodes were fabricated at the tip of the curved surface of a hypodermic needle employing spray coating and photolithography with a film photomask. First, SiO_2_ was deposited on the needle for the insulation layer using low-pressure chemical vapor deposition (LPCVD). The electrodes were then fabricated on the insulation layer of SiO_2_, followed by selective patterning of the photoresist of SU-8 (2000.5, low viscosity). As shown in Figure 1b, the electrodes were successfully fabricated on the needle. The overall length and width of the fabricated IDEs were 300 μm and 400 μm, respectively. Both the width and gap of the IDEs were as small as 20 μm, which is close to the smallest dimension available in commercial film photomask. Figure 1c shows the SPL of the photoresist on the needle insulated by SiO_2_.

### 2.3. Experimental Setup

Before the experiments using PBS and porcine tissues, a durability test was conducted by piecing the device into PDMS (polydimethylsiloxane) elastomer with a 1:10 ratio of hardener to resin. The needle was repeatedly inserted into the PDMS until the insulation layer, passivation layer, and/or the electrodes peeled off. The status of the electrodes, insulation, and passivation layer were monitored through a microscope to detect the moment of peeling or disconnection. The passivation layer of SU-8 and electrodes on the insulation layer of SiO_2_ were well preserved even after 50 insertions into the PDMS, whereas the passivation layer of Parylene C and electrodes on the insulation layer of Parylene C could endure only 20 insertions.

**Figure 2:**
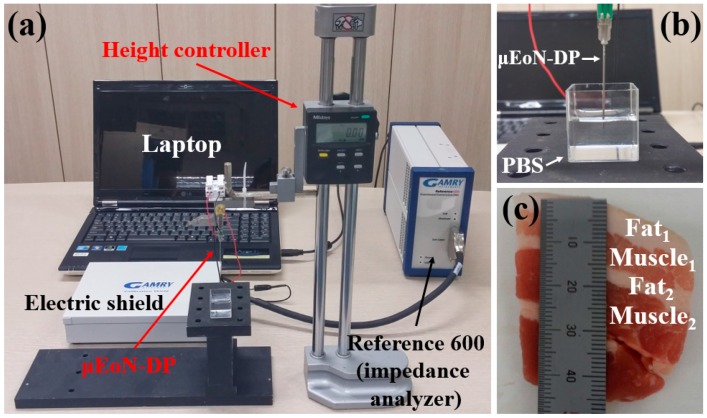
Images of experimental setup: (**a**) overall setup; (**b**) μEoN-DP immersed in the PBS; and (**c**) four-layered porcine tissue.

In order to evaluate the discrimination capability of the μEoN-DP, the electrical impedances of PBS were measured at various concentration levels over the frequency range from 100 Hz to 1 MHz (the maximum measurable range of impedance analyzer, Gamry Instruments, reference 600). The frequency range was decided as from 100 Hz to 1 MHz because the electrode polarization typically induced below 100 Hz severely influences the impedance measurements [32,33,34,35,36]. The operating voltage was selected as 100 mV_rms_ to minimize the damage to cells or tissues, taking into consideration future trials, such as tissue sampling and surgery [37]. Note that the maximum value of Johnson noise at the highest frequency in the measurement (1 MHz) can be estimated to be as small as 12.49 μV_rms_ [38], which was negligibly small compared to the applied voltage of 100 mV_rms_. As shown in Figure 2a,b, the μEoN-DP is fixed on the height controller with a resolution of 10 μm. The part connected between the copper wire extended from the μEoN-DP and the shielded cable of the impedance analyzer was placed in a Faraday shield (Gamry Instruments) to minimize the external noise. Other electronic and mechanical equipment were positioned sufficiently apart from the measurement place. The various concentration levels of PBS were prepared as 1×, 0.5×, 0.25×, 0.125×, and 0.0625× by serially diluting the 1× PBS (Life technologies, pH: 7.4, osmolality: 280–315 mOsm/kg) with deionized water (1:1 mixing ratio). The penetration depth of the μEoN-DP into the PBS was maintained at 5 mm for consistency of the experimental conditions. Prior to the measurements, the μEoN-DP was discharged by immersing the device in the samples for 30 s to eliminate noise. After evaluation of the discrimination capability of the μEoN-DP, depth profiling will be performed using four-layered (fat_1_–muscle_1_–fat_2_–muscle_2_) porcine tissue (Figure 2c) to verify the effectiveness of the discrimination capability of the μEoN-DP for biotissues. While the μEoN-DP sequentially penetrated the four-layered porcine tissue (fat_1_–muscle_1_–fat_2_–muscle_2_), the electrical impedances were measured with a penetration interval of 1mm. The cleaning process could be conducted using acetone, deionized water, and air drying using a heat gun (150 °C) because the SU-8 passivation layer has a strong chemical resistance. The room temperature and humidity was maintained at 23 °C and 40%, respectively.

## 3. Results and Discussion

### 3.1. PBS at Various Concentration Levels

**Figure 3:**
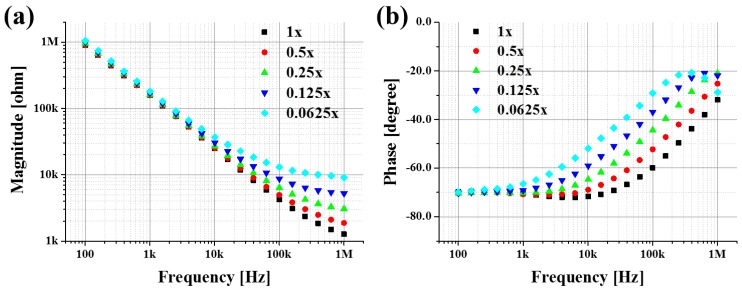
Averaged electrical impedances of PBS at various concentration levels: (**a**) magnitude and (**b**) phase.

In order to verify the discrimination capability of the μEoN-DP, electrical impedances of PBS at various concentration levels were measured. The electrical impedance of each concentration level was measured 10 times, and their average values were depicted in Figure 3. The standard deviations of the magnitude and phase were less than 2.23% and 0.82%, respectively, for every concentration level and at all the investigated frequencies showing a high discrimination capability.

For the magnitude, differences among each concentration level of PBS were observed at higher frequencies, and those for the phase were observed at mid-range frequencies. The high impedance at low frequencies can be explained by the capacitive property of the double layer, which can be modeled as a CPE (constant phase element) [39]. The CPE impedance is expressed by *Z* = 1/{*Y*_0_·(*jω*)*^n^*}, whose parameters will be extracted through curve fitting.

In the majority of frequency range, as the concentration levels of PBS decreased, the magnitude and the phase increased. The larger real (*Z*′) part of the impedance was observed in the lower concentration levels of PBS, which indicate that the conductivity becomes lower as concentration levels of PBS decrease. The strong dependency of the real part of the impedance on the frequency can be attributed predominantly to the parallel connection of the resistance (R) and capacitance (C). From Figure 3, as the concentration decreased, the absolute values of the real and imaginary parts of the impedance increased, and their ratio became larger. Thus, the phase values at low concentration levels of PBS were larger than those at high concentration levels. As the frequency increased, the real part of the impedance became more dominant. At low concentration levels of 0.125× and 0.0625×, the increasing tendency of the phase reversed after 400 kHz and 630 kHz, respectively, because the capacitance decrease influences the imaginary part of the impedance more than the frequency increase does.

From the experimental results of the sensor output, the μEoN-DP was verified to discriminate dissimilar materials having different electrical properties. In order to quantify the extent of the discrimination capability of the μEoN-DP, discrimination index (*DI*), which will be employed in the next section for biotissue discrimination, can be defined as follows:

(1) $$DI=\overset{4}{\underset{i=1}{\sum }}DI_{i}=\overset{4}{\underset{i=1}{\sum }}\left|C_{i}-C_{i+1}\right|/\left(D_{i}+D_{i+1}\right)$$

**Figure 4:**
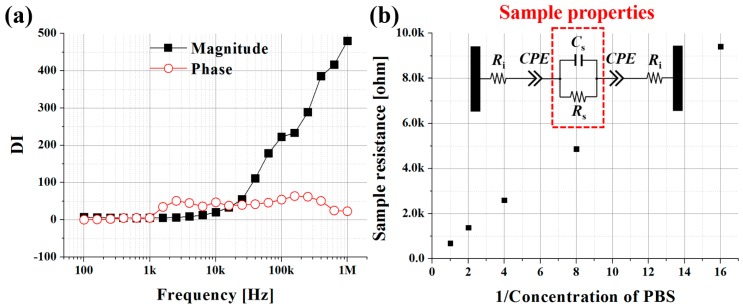
(**a**) Discrimination index (*DI*) to find the optimal frequency at which the discrimination capability of μEoN-DP was maximized; and (**b**) the linearity of extracted sample resistance, along with the reciprocal value of PBS concentration. The inset represents the electrical equivalent circuit to extract the resistance (*R*_s_), capacitance (*C*_s_), and CPE parameters (*Y*_0_ and *n*).

The variables (*C*, *D*) and subscript (i) represent the mean impedance value, standard deviation, and concentration level of PBS (1:0.0625×, 2:0.125×, 3:0.25×, 4:0.5×, and 5:1×), respectively. The *DI* value, obtained at all investigated frequencies, becomes larger as the difference of the mean values in the numerator increases and/or the sum of the standard deviations in the denominator decreases. The difference of electrical impedance between two concentration levels of PBS was divided by the sum of their standard deviations to normalize the *DI* value. The *DI*s (*DI*_1_, *DI*_2_, *DI*_3_, and *DI*_4_) were then summed and depicted, as shown in Figure 4a. The optimal frequencies at which the discrimination capability can be maximized for the magnitude and phase were found at 1 MHz and 150 kHz, respectively; these are the frequencies at which the experimental curves in Figure 3 are best distinguishable.

Although we evaluated the discrimination capability of the μEoN-DP using PBS at various concentration levels, the experimental results still included the device resistance and sample-device interactions, such as charge transfer resistance and double layer capacitance. These interactions should be minimized from the sensor output to obtain only the sample resistance and the capacitance by curve fitting with the equivalent circuit shown in Figure 4b. The subscripts i and s stand for internal and sample, respectively. *C*_s_ (the sample capacitance) is connected in parallel with *R*_s_ (the sample resistance), and they are in series with *R*_i_ (the internal resistance) and the CPE. The internal resistance was experimentally confirmed as approximately 100 Ω. The CPE was placed in an equivalent circuit to reflect the double layer capacitance, charge transfer resistance, electrode polarization, and rough surface of the electrodes [39]. The extracted sample resistance (*R*_s_), sample capacitance (*C*_s_), and CPE parameters (*Y*_0_ and *n*) are summarized in Table 1.

As the concentration decreased, the resistances increased, whereas the capacitance decreased. As shown in Figure 4b, the sample resistances (*R*_s_) were inversely proportional and roughly linear to the decrease in concentration levels because the conductivity of deionized water can be assumed to be extremely low; therefore, the conductivity of the 1:1 mixture of 1× PBS and deionized water became half the conductivity of 1× PBS. However, the contribution of deionized water will not be negligible, as concentration significantly decreases. The extracted resistance and capacitance were in sufficient agreement with those theoretically estimated based on the mixing ratio of PBS and deionized water; discrepancies were less than 8.02% and 1.85%, respectively. As the concentration decreased, the influence of the CPE on the sensor output also decreased. At a concentration of 1×, the CPE impedance accounted for 99.9% at 100 Hz and 29.6% at 1 MHz in the capacitive effect on the sensor output, which decreased to 99.1% and 5.7% at the same frequencies at a concentration of 0.0625×.

In order to discuss the advantage of micro EIS over macro EIS, their specific electrical properties were investigated in terms of sensitivity, based on the equivalent circuit and extracted parameters from PBS experiments. "Microsized" is derived from the macro by miniaturizing the dimensions of the electrodes, whereas their effective sensing areas are kept constant. In a microscale, each magnitude contributed by the sample resistance (*R*_s_) and capacitance (*C*_s_) is smaller than that in a the macroscale, which was verified using a COMSOL simulation (COMSOL, Inc., USA). Thus, the sensitivity in a microscale tends to be larger than that in a macroscale, assuming that the same amount of perturbation in the magnitude of electrical impedance is given to the sample. This tendency becomes more prominent at high frequencies, which can explain the reason why the highest *DI* for the magnitude was found at 1 MHz.

### 3.2. Depth Profiling Into Four-Layered Porcine Tissue

In order to evaluate the effectiveness of the discrimination capability of the μEoN-DP for biotissues, depth profiling was conducted using four-layered (fat_1_–muscle_1_–fat_2_–muscle_2_) porcine tissue. Depth profiling was conducted up to 25 mm with a penetration interval of 1 mm. As shown in Figure 2c, the fat_1_ (8 mm), muscle_1_ (7 mm), fat_2_ (3 mm), and muscle_2_ (7 mm) tissues were arranged in order within 25 mm of the porcine tissue.

**Figure 5:**
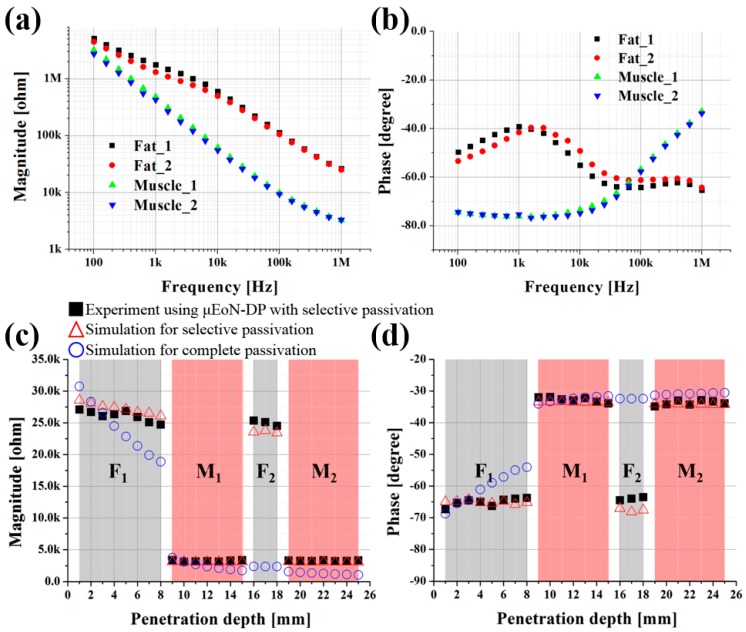
Mean impedance of each layer in four-layered porcine tissue: (**a**) magnitude and (**b**) phase; depth profiling and simulation results obtained at the optimal frequency for four-layered porcine tissue: (**c**) magnitude and (**d**) phase (simulations were carried out to verify the advantage of selective passivation over complete passivation in terms of sensor output).

The impedances measured in the same layer of porcine tissue were averaged, as shown in Figure 5a,b. For the magnitude and phase, the maximum standard deviations of fat_1_ tissue were 17.53% at 1.57 kHz and 6.55% at 6.33 kHz, respectively. The values for the muscle_1_ tissue were 6.26% and 3.87%, both at 998 Hz, respectively. Fat_2_ and muscle_2_ showed similar extents of standard deviations. From the experimental impedances, it was confirmed that the magnitudes of fat tissue were higher than those of muscle tissues at all the investigated frequencies. This implies that more electrical current can flow through muscle tissues with less resistance, which was in good agreement with the reported study [40].

To suggest the best discrimination results of depth profiling considering real-time applications for practical use, it is necessary to find the optimal frequency at which the discrimination capability is maximized. In order to find the optimal frequency, the *DI* was evaluated for the porcine tissues. The *DI*_ij_ for two of the dissimilar tissues is defined as the ratio of the absolute value of mean difference and the sum of the standard deviations, which is shown in Equation (2) as a generalized expression. The variables (*C*, *D*, and *N*) represent the mean impedance value, standard deviation and the total number of tissues, respectively. The subscripts (f, m, i, and j) represent fat tissue, muscle tissue, i*th* tissue, and j*th* tissue, respectively.

(2) $$DI=\sum_{i=1}^{N_{f}}\sum_{j=1}^{N_{m}}DI_{\mathrm{ij}}=\sum_{i=1}^{N_{f}}\sum_{j=1}^{N_{m}}\left|C_{f,i}-C_{m,j}\right|/\left(D_{f,i}+D_{m,j}\right)$$

The *DI* basically includes three values of *DI_ij_* for three physical boundaries within 25 mm in the porcine tissue. Still, there is the fourth possible combination of dissimilar tissues, i.e., fat_1_ and muscle_2_, which is necessary to completely reflect the symmetric properties of *DI*. Thus, further comparison between fat_1_ and muscle_2_ tissues was also included in the definition of *DI*, even if there is no physical boundary between them. The optimal frequency was determined as the frequency at which sum of the four *DI*_ij_ was maximized. The magnitude differences between dissimilar tissues seem to be larger at low frequencies around 5 kHz compared to those at higher frequencies. However, the largest *DI*s for the magnitude and phase (271.51 and 98.03, respectively) were found both at 1 MHz because the mean difference values were divided into sum of the standard deviations in the definition of *DI*. It should be noted that the small value of the denominator implies small deviation, indicating a high repeatability of the experimental results.

The experimental results at the optimal frequency of 1 MHz were plotted as shown in Figure 5c,d with respect to the penetration depth. For both the magnitude and the phase, there were significant differences between dissimilar tissues at the optimal frequency. However, slight discrepancies were observed between the similar tissues of fat_1_ and fat_2_, which will be investigated using a 3D COMSOL simulation in the next section.

### 3.3. Simulation Verification of Selective Passivation Layer

In order to verify the advantage of selective passivation over complete passivation in terms of the sensor output, the following two 3D COMSOL simulations were conducted: (a) only the sensing IDEs with SPL were exposed to biotissues; and (b) the entire electrode (IDEs and connection lines) with CPL were exposed to biotissues. This will also reveal the reason for the slight differences in the electrical impedance between fat_1_ and fat_2_ tissues. In the simulation, the material properties of fat_1_ and fat_2_ tissues were assumed to be identical in order to investigate only the influence of connection lines. As shown in Figure 5c,d, the μEoN-DP with SPL showed clear discrimination results compared to the results with CPL.

For the case with the CPL, the magnitude decreased and the phase increased along with the penetration depth of the electrodes, which implies that the immersed connection lines in the biotissues considerably influenced the sensor output. The total output impedance becomes smaller as the penetration proceeds into the biotissues. This can be explained by the fact that the influence of the connection lines on the sensor output increases because the impedance of the connection lines and sensing IDEs are electrically connected in parallel. This decreasing tendency in total output impedance was more prominent in fat_2_ tissues compared to that in other tissues. The magnitude and phase of the fat_2_ tissues are similar to those of the muscle tissues, which makes it difficult to discriminate even between dissimilar tissues. Thus, a post-compensation process that can minimize the unwanted error induced from the connection lines is required to obtain more accurate results.

For the case with SPL, although the fat_1_ and fat_2_ tissues were assumed to have the identical electrical properties, a slight decrease in magnitude and increase in phase were observed in the simulation results as the penetration depth of the electrodes increased in the fat tissues. The slight discrepancies between the fat_1_ and fat_2_ tissues were also observed through simulations. The simulation results were in sufficient agreement with the experimental ones; the discrepancies were as small as 6.02% for both magnitude and phase. It is evident from the simulation and experimental results that the impedance variation in each tissue and discrepancies between fat_1_ and fat_2_ tissues can be accounted for the influence of connection lines on the sensor output; the SPL cannot perfectly block the alternating current through the connection lines. In the muscle tissues, the influence of the connection lines on the sensor output appeared to be negligible compared to that in the fat tissues. This can be explained by the fact that electric fields induced from the connection lines faces greater difficulty in passing through the muscle tissues than in passing through fat tissues. This is because the conductivity of muscle tissues is several hundred times higher than that of fat tissues, which implies that the muscle tissues can confine more electric fields within the SPL than fat tissues can. The dependencies of the magnitude on the type of passivation layer are listed in Table 2.

With the help of the SPL, the relative influence of the connection lines on the sensor output can be effectively minimized without any post-compensation process. The magnitude decreased by 38.59% with the CPL as the penetration depth increased in the fat_1_ tissue, while the magnitude decreased only slightly by 8.70% with the SPL. In addition, the averaged magnitude with CPL is estimated through simulation to decrease by 90.14% between the fat_1_ and fat_2_ fat tissues, whereas the averaged magnitude with the SPL was experimentally confirmed to decrease by only 4.25%.

These results suggest that the μEoN-DP could clearly discriminate between dissimilar tissues so that the maximum depth of buried tumors during partial nephrectomy can be located to reduce the positive surgical margin ratio. In the case of laparoscopic surgery, an iatrogenic organ injury caused by Veress needle insertion can be promptly detectable, enabling immediate management to reduce perioperative complications. In addition, the μEoN-DP can be utilized as a spinal needle capable of locating the exact position for drug delivery during spinal or epidural anesthetic procedures.

## 4. Conclusions

In conclusion, the μEoN-DP having a SPL on IDEs was newly designed to more accurately measure the electrical impedance in the biotissues up to 25 mm in depth by reducing the relative influence of the connection lines on the sensor output. Firstly, the discrimination capability of the μEoN-DP was verified by using PBS at various concentration levels. The extracted resistance and capacitance were in good agreement with those theoretically estimated based on the mixing ratio of PBS and deionized water; discrepancies were less than 8.02% and 1.85%, respectively. We believe that those discrepancies can be minimized by conducting the experiment in an electromagnetically shielded room and replacing the copper wire with a shielded cable. Secondly, to evaluate the effectiveness of the discrimination capability of the μEoN-DP for biotissues, the electrical impedance of four-layered porcine tissue was measured with respect to the penetration depth. For both the magnitude and the phase, the optimal frequency for the best discrimination was found at 1 MHz. Finally, through 3D COMSOL simulations, it was confirmed that the SPL can effectively reduce the unwanted error induced from the connection lines on the sensor output without a post-compensation process. In order to additionally improve the discrimination capability of the μEoN-DP, the impedance ratio of the connection lines and the sensing IDEs should be larger, reducing the relative influence of the connection lines on the sensor output by increasing the effective sensing area.

## Figures and Tables

**Table 1:** Extracted resistance (*R*_s_), capacitance (*C*_s_), and parameters (*Y*_0_ and *n*) of CPE through curve fitting with the experimental results.

Concentration	*R*_s_ [kΩ]	*C*_s_ [pF]	*Y*_0_ [S·s^n^]	*n*
1×	0.689	26.71	12.72 × 10^−9^	0.793
0.5×	1.375	17.26	13.33 × 10^−9^	0.787
0.25×	2.597	12.58	13.92 × 10^−9^	0.777
0.125×	4.874	10.08	14.34 × 10^−9^	0.768
0.0625×	9.413	8.74	14.92 × 10^−9^	0.755

**Table 2:** Dependency of the magnitude on the type of passivation layer: A is the decreasing rate of the magnitude in the fat_1_ tissue and B is the decreasing rate of the averaged magnitude between the fat_1_ and fat_2_ tissues.

Passivation Type	A	B
SPL	8.70%	4.25%
CPL	38.59%	90.14%
